# A history of hybrids? Genomic patterns of introgression in the True Geese

**DOI:** 10.1186/s12862-017-1048-2

**Published:** 2017-08-22

**Authors:** Jente Ottenburghs, Hendrik-Jan Megens, Robert H. S. Kraus, Pim van Hooft, Sipke E. van Wieren, Richard P. M. A. Crooijmans, Ronald C. Ydenberg, Martien A. M. Groenen, Herbert H. T. Prins

**Affiliations:** 1 Resource Ecology Group, Wageningen University & Research, Droevendaalsesteeg 3a, 6708 PB Wageningen, the Netherlands; 2 Animal Breeding and Genomics, Wageningen University & Research, Droevendaalsesteeg 1, 6708 PB Wageningen, the Netherlands; 3 Department of Migration and Immuno-Ecology, Max Planck Institute for Ornithology, Am Obstberg, 1D-78315 Radolfzell, Germany; 4 Department of Biology, University of Konstanz, D-78457 Constance, Germany; 5 Centre for Wildlife Ecology, Simon Fraser University, V5A 1S6, Burnaby, BC Canada

**Keywords:** Hybridization, Phylogenetic Networks, D-statistic, PSMC, Phylogenomics

## Abstract

**Background:**

The impacts of hybridization on the process of speciation are manifold, leading to distinct patterns across the genome. Genetic differentiation accumulates in certain genomic regions, while divergence is hampered in other regions by homogenizing gene flow, resulting in a heterogeneous genomic landscape. A consequence of this heterogeneity is that genomes are mosaics of different gene histories that can be compared to unravel complex speciation and hybridization events. However, incomplete lineage sorting (often the outcome of rapid speciation) can result in similar patterns. New statistical techniques, such as the D-statistic and hybridization networks, can be applied to disentangle the contributions of hybridization and incomplete lineage sorting. We unravel patterns of hybridization and incomplete lineage sorting during and after the diversification of the True Geese (family Anatidae, tribe Anserini, genera *Anser* and *Branta*) using an exon-based hybridization network approach and taking advantage of discordant gene tree histories by re-sequencing all taxa of this clade. In addition, we determine the timing of introgression and reconstruct historical effective population sizes for all goose species to infer which demographic or biogeographic factors might explain the observed patterns of introgression.

**Results:**

We find indications for ancient interspecific gene flow during the diversification of the True Geese and were able to pinpoint several putative hybridization events. Specifically, in the genus *Branta*, both the ancestor of the White-cheeked Geese (Hawaiian Goose, Canada Goose, Cackling Goose and Barnacle Goose) and the ancestor of the Brent Goose hybridized with Red-breasted Goose. One hybridization network suggests a hybrid origin for the Red-breasted Goose, but this scenario seems unlikely and it not supported by the D-statistic analysis. The complex, highly reticulated evolutionary history of the genus *Anser* hampered the estimation of ancient hybridization events by means of hybridization networks. The reconstruction of historical effective population sizes shows that most species showed a steady increase during the Pliocene and Pleistocene. These large effective population sizes might have facilitated contact between diverging goose species, resulting in the establishment of hybrid zones and consequent gene flow.

**Conclusions:**

Our analyses suggest that the evolutionary history of the True Geese is influenced by introgressive hybridization. The approach that we have used, based on genome-wide phylogenetic incongruence and network analyses, will be a useful procedure to reconstruct the complex evolutionary histories of many naturally hybridizing species groups.

**Electronic supplementary material:**

The online version of this article (doi:10.1186/s12862-017-1048-2) contains supplementary material, which is available to authorized users.

## Background

The impacts of hybridization on the process of speciation are manifold [1]. Hybridization may slow down or even reverse species divergence. It may also accelerate speciation via adaptive introgression or contribute to species diversity through the formation of new hybrid taxa. These diverse effects occur at different spatial scales and during different stages across the speciation continuum [2]. The consequences of hybridization and its role in impeding or promoting speciation are thus expected to vary widely among hybridizing taxa and at different stages of divergence. In every case, the pattern of hybridization is only a single snapshot of a complex and continuously changing interaction.

Genomics has become a standard practise, also in ornithology [3, 4], opening avenues to answer longstanding questions in speciation and hybridization [2, 5]. Studies in speciation and hybridization genomics revealed that levels of genetic differentiation between species can be highly variable across the genome: genetic differentiation accumulates in certain genomic regions, while divergence is hampered in other regions by homogenizing gene flow, resulting in a heterogeneous genomic landscape [6–8]. A consequence of this heterogeneity is that genomes are mosaics of different gene histories [9–11] that can be compared to unravel complex speciation and hybridization events [12, 13].

Complex evolutionary histories with rapid speciation (leading to incomplete lineage sorting) and hybridization mostly result in high levels of phylogenetic incongruence (i.e. gene tree discordance), which can be difficult to capture in a traditional, bifurcating phylogenetic tree. Phylogenetic networks can be a powerful tool to display and analyse these evolutionary histories [14, 15]. For example, Suh et al. [16] quantified the amount of incomplete lineage sorting along the Neoaves phylogeny [17] using presence/absence data for 2118 retrotransposons and concluded that the “complex demographic history [of the Neoaves] is more accurately represented as local networks within a species tree.”

Here, we study patterns of hybridization and incomplete lineage sorting during and after the diversification of the True Geese (Table 1), a group of naturally hybridizing bird species [18, 19]. The True Geese are classified in the waterfowl tribe Anserini and have been traditionally divided over two genera: *Anser* and *Branta* [20]. Hybrids have been reported within each genus [21–27], but also intergeneric hybrids have been documented [28–31]. Previous studies suggested that the evolutionary history of the True Geese is heavily influenced by hybridization and rapid diversification [12, 32]. In this study, we explore this suggestion using a network approach and taking advantage of phylogenetic incongruence across the whole genome by fully re-sequencing all species of the True Geese clade. Moreover, we attempt to quantify the relative contributions of gene flow and incomplete lineage sorting during the evolution of this bird group.

**Table 1:** Current taxonomy for the True Geese (tribe Anserini), previously published in Ottenburghs et al. (2016)

English Name	Scientific Name	Subspecies
Genus ANSER		
Swan Goose	*Anser cygnoides*	
Taiga Bean Goose	*Anser fabalis*	*A. f. fabalis* *A. f. johanseni* *A. f. middendorffii*
Tundra Bean Goose	*Anser serrirostris*	*A. s. rossicus* *A. s. serrirostris*
Pink-footed Goose	*Anser brachyrhynchus*	
Greater White-fronted Goose	*Anser albifrons*	*A. a. albifrons* (Eurasian) *A. a. flavirostris* (Greenland) *A. a. gambeli* (Western) *A. a. frontalis* (Western) *A. a. elgasi* (Tule)
Lesser White-fronted Goose	*Anser erythropus*	
Greylag Goose	*Anser anser*	*A. a. anser* (European) *A. a. rubrirostris* (Siberian)
Bar-headed Goose	*Anser indicus*	
Emperor Goose	*Anser canagicus*	
Snow Goose	*Anser caerulescens*	*A. c. caerulescens* *A. c. atlantica*
Ross’ Goose	*Anser rossii*	
Genus BRANTA		
Brent Goose	*Branta bernicla*	*B. b. bernicla* (Dark-bellied) *B. b. hrota* (Pale-bellied or Atlantic) *B. b. nigricans* (Black) *B. b. orientalis*
Barnacle Goose	*Branta leucopsis*	
Cackling Goose	*Branta hutchinsii*	*B. h. leucopareia* (Aleutian) *B. h. hutchinsii* (Richardson’s) *B. h. minima* (Minima) *B. h. taverneri* (Taverner’s)
Canada Goose	*Branta canadensis*	*B. c. moffitti* *B. c. maxima* *B. c. occidentalis* *B. c. fulva* *B. c. canadensis* *B. c. interior* *B. c. parvipes*
Hawaiian Goose	*Branta sandvicensis*	
Red-breasted Goose	*Branta ruficollis*	

The contrasting evolutionary histories of these closely related genera also provide an excellent opportunity to study the effects of hybridization on the speciation process. The *Anser*-clade can be regarded as an adaptive radiation and was probably affected more by hybridization compared to the more gradually diversifying *Branta*-clade [12]. Therefore, we hypothesize that the phylogenetic network of *Anser* will be more complex (i.e. contain more interconnections between the taxa) compared to the *Branta*-network. Moreover, statistics quantifying interspecific gene flow, such as the D-statistic [33], are expected to be higher for *Anser* compared to *Branta*.

## Methods

### Genomic dataset

We collected blood samples from 19 goose (sub)species (Additional file 1: Table S1). From these blood samples, genomic DNA was isolated using the Qiagen Gentra kit (Qiagen Inc.). DNA quantity and quality were assessed using Qbit (Invitrogen, Life Technologies). Sequence libraries were made following Illumina protocols and sequenced paired-end (100 bp) on the HiSeq2500 (Illumina Inc.).

Paired-end reads were mapped to the Mallard (*Anas platyrhynchos*) genome, version 73 [34] using SMALT (http://www.sanger.ac.uk/science/tools/smalt-0). Over 99% of the reads mapped successfully in all samples, but to decrease the incidence of off-site mapping only properly mapped reads were accepted, leading to mapping rates between 63% and 78% (Additional file 2: Table S2). Next, duplicate sequences were removed using SAMtools-dedup [35] and realigned with IndelRealigner in GATK 2.6 [36]. Variant sites calling was performed using UnifiedGenotyper in GATK 2.6 [36] with a heterozygosity value of 0.01 and a minimum base quality of 20. Heterozygous sites were coded following the IUPAC nucleotide codes (e.g., R for A and G). The genomic positions for exons that were one-to-one orthologous between Mallard and other bird species (chicken, turkey, flycatcher and zebra finch) were retrieved from the ENSEMBL database.

From whole genome sequence data, we thus filtered out high quality exonic sequences. The final dataset is comprised of 41,736 unique exons, representing 5887 genes. The total alignment (6,630,626 bp) was used in the neighbour-joining network and D-statistics analyses described below. In addition, we selected 3570 one-to-one orthologous genes with a minimum length of 500 bp. These genes were analysed separately under a GTR + Γ substitution model with 100 rapid bootstraps in RAxML 8.3 [37, 38]. The resulting gene trees were filtered on average bootstrap support (minimum >50). This final set of 3558 well-supported gene trees was used in the analysis to construct hybridization networks – which calculate evolutionary trees taking into account hybridization – and to determine the timing of gene flow. Ottenburghs et al. [19] provide the phylogenetic framework for the current study, which focuses on introgressive hybridization during the evolutionary history of the True Geese.

### Gene flow analysis

The D-statistic is a statistical test that was first employed to quantify the amount of genetic exchange between Neanderthals and humans [39]. It exploits the asymmetry in frequencies of two nonconcordant gene trees in a three-population setting [33]. Consider three populations (P1, P2 and P3) and an outgroup (O), of which P1 and P2 are sister clades. In this ordered set op populations [P1, P2, P3, O], two allelic patterns are of interest: “ABBA” and “BABA”. The pattern ABBA refers to the situation in which P1 has the outgroup allele “A” and P2 and P3 share the derived allele “B”, while the pattern BABA refers to the situation in which P2 has the outgroup allele “A” and P1 and P3 share the derived allele “B”. Under the null hypothesis that P1 and P2 are more closely related to each other than to P3, and if the ancestral populations of P1, P2, P3 were panmictic, then it is expected that the derived alleles in P3 match the derived alleles in P1 and P2 equally often [40, 41]. In other words, the patterns ABBA and BABA should occur in equal frequencies and the D-statistic should equal zero:

$$ D\left({P}_1,{P}_2,{P}_3,O\right)=\frac{\sum_{i=1}^n{C}_{ABBA}(i)-{C}_{BABA}(i)}{\sum_{i=1}^n{C}_{ABBA}(i)+{C}_{BABA}(i)} $$

A D-statistic equal to zero is expected under incomplete lineage sorting. Gene flow between P1 and P3 (indicated by an overrepresentation of BABA) or P2 and P3 (indicated by an overrepresentation of ABBA) result in a D-statistic that is significantly different from zero. For both genera, D-statistics were calculated for all possible combinations of three species in the program HybridCheck version 1.0.1 [42]. We combined all species of the other genus as the outgroup. To test for significance, we performed jackknife resampling using blocks of 50,000 bp. We did not quantify asymmetric gene flow between genera due to the lack of a proper outgroup.

**Fig. 1:**
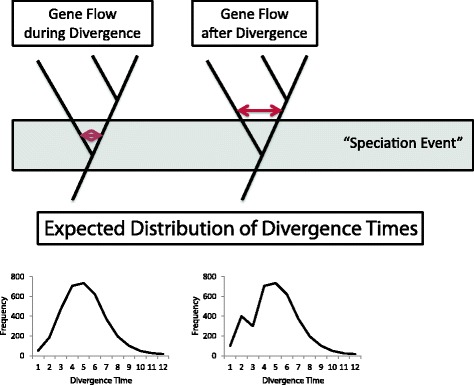
Expected distribution of divergence times. If gene flow occurred during the diversification process, it will be indistinguishable from genetic divergence at other loci, resulting in a single peak (left graph). A similar pattern is expected under incomplete lineage sorting, but to discriminate between gene flow and incomplete lineage sorting, other analyses are warranted. If, on the other hand, gene flow occurred after the diversification process, introgressed loci will show more recent divergence times, which becomes apparent as a recent, smaller peaks (right graph)

To infer the timing of gene flow (during or after the diversification), we dated 3558 gene trees using the software PATHd8 version 1.0 [43], setting the divergence time between the genera at 9.5 million years ago (based on previous estimates, [44, 45]). For every species pair, histograms were constructed from the resulting divergence times [46]. The patterns expected under incomplete lineage sorting and when gene flow occurred during or after the diversification are presented in Fig. 1.

### Network analyses

A previous phylogenomic analysis of the True Geese indicated high levels of gene tree discordance, which can be caused by hybridization and/or incomplete lineage sorting [12]. To visualize this phylogenetic incongruence, we constructed a phylogenetic neighbour-joining network using the ordinary least squares method (with default settings) in SplitsTree version 4.1.4.2 [15]. This network was based on genetic distances, which were calculated in RAxML 8.3 with a GTR + Γ substitution model [12, 37]. We calculated the degree distributions (i.e. the number of connections for each node in a network) for each genus to quantify the complexity of the networks using the R-package igraph [47]. The degree distributions for each genus were compared by means of a general linear model with Poisson distribution in R version 3.2.2.

Hybridization networks are networks that attempt to reconstruct a phylogenetic tree with the fewest amount of hybridization events [15, 48]. For each genus, we combined 3558 gene trees into hybridization networks using the Autumn algorithm [49] with default settings in Dendroscope version 3.4.4 [50].

### Demographic analysis

We conducted a demographic analysis using a hidden Markov model approach as implemented in the software package PSMC [51]. A consensus sequence was generated from BAM files using the ‘pileup’ command in SAMtools [35]. For the PSMC analyses, we used the parameter settings suggested by Nadachowska-Brzyska et al. [52], namely “N30 –t5 –r5 –p 4 + 30*2 + 4 + 6 + 10.”

## Results

### Gene flow analysis

**Fig. 2:**
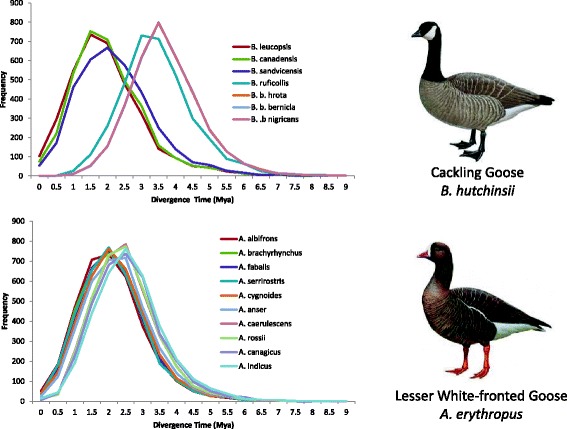
Distribution of divergence times for Lesser White-fronted Goose with all *Anser* species and for Cackling Goose with all *Branta* species. All distributions show a single peak, indicating gene flow during divergence. The divergence time of several gene trees was close to zero, suggesting low levels of recent gene flow between certain species

The D-statistic analysis supported gene flow between several goose species (Table 2 Additional file 5: Table S3). Although the D-statistics for *Anser* were slightly higher compared to *Branta*, there was no significant difference (Mann Whitney U, W = 4659, *p* = 0.088). To infer the timing of gene flow (during or after the diversification), we took advantage of gene tree discordance and constructed histograms based on divergence times of 3558 gene trees. All analyses supported a scenario of gene flow during divergence with low levels of recent gene flow because the histograms all displayed one peak corresponding to the initial species split. The divergence time of several gene trees was close to zero, suggesting low levels of recent gene flow between certain species. Figure 2 shows two examples, involving the Cackling Goose and the Lesser White-fronted Goose (for other species, see Additional file 3: Figure S1).

**Table 2:** Significant D-statistics (Z-score > 3 and *p* < 0.0001) for different combination of three goose species

P1	P2	P3	D-statistics	Z-score
BRANTA				
Barnacle Goose	Cackling Goose	Canada Goose	0.087	4.322
Barnacle Goose	Cackling Goose	Red-breasted Goose	0.146	6.022
Barnacle Goose	Cackling Goose	Black Brent	0.094	3.516
Barnacle Goose	Canada Goose	Red-breasted Goose	0.1	4.037
Barnacle Goose	Hawaii Goose	Red-breasted Goose	0.094	4.192
Barnacle Goose	Black Brent	Red-breasted Goose	0.073	3.022
ANSER				
Lesser White-fronted	Greater White-fronted	Pink-footed Goose	0.126	6.116
Taiga Bean Goose			0.09	4.413
Pink-footed Goose	Greater White-fronted	Emperor Goose	0.088	3.701
Tundra Bean Goose			0.079	3.383
Taiga Bean Goose			0.128	5.457
Greylag Goose			0.079	3.537
Taiga Bean Goose	Greater White-fronted	Bar-headed Goose	0.083	3.470
Greater White-fronted	Lesser White-fronted	Swan Goose	0.114	5.279
Pink-footed Goose			0.15	6.821
Tundra Bean Goose			0.16	8.15
Taiga Bean Goose			0.173	8.385
Greater White-fronted	Lesser White-fronted	Ross’ Goose	0.104	5.325
Pink-footed Goose			0.12	5.421
Tundra Bean Goose			0.103	4.682
Taiga Bean Goose			0.083	3.93
Swan Goose			0.103	3.984
Emperor Goose			0.102	4.51
Greater White-fronted	Lesser White-fronted	Snow Goose	0.165	7.336
Pink-footed Goose			0.174	7.032
Tundra Bean Goose			0.157	7.053
Taiga Bean Goose			0.137	6.224
Swan Goose			0.156	6.525
Greylag Goose			0.086	4.054
Emperor Goose			0.14	6.007
Pink-footed Goose	Lesser White-fronted	Emperor Goose	0.076	3.164
Tundra Bean Goose			0.07	3.0
Taiga Bean Goose			0.12	5.419
Greylag Goose			0.078	3.472
Taiga Bean Goose	Pink-footed Goose	Bar-headed Goose	0.082	3.436
Greater White-fronted	Greylag Goose	Ross’ Goose	0.087	3.719
Pink-footed Goose			0.107	4.496
Tundra Bean Goose			0.086	3.408
Swan Goose			0.084	3.049
Emperor Goose			0.163	6.67
Greater White-fronted	Greylag Goose	Snow Goose	0.072	3.055
Pink-footed Goose			0.091	3.416
Emperor Goose			0.139	5.59
Emperor Goose	Tundra Bean Goose	Ross’ Goose	0.078	3.692
Emperor Goose	Tundra Bean Goose	Snow Goose	0.074	3.317
Emperor Goose	Taiga Bean Goose	Ross’ Goose	0.139	6.346
Emperor Goose	Taiga Bean Goose	Snow Goose	0.139	6.655
Taiga Bean Goose	Greylag Goose	Bar-headed Goose	0.09	4.059

Asymmetric gene flow is between P2 and P3. The outgroup for *Branta* species was a consensus sequence based on all *Anser* species, while the outgroup for *Anser* species was a consensus sequence based on all *Branta* species

### Network analyses

**Fig. 3:**
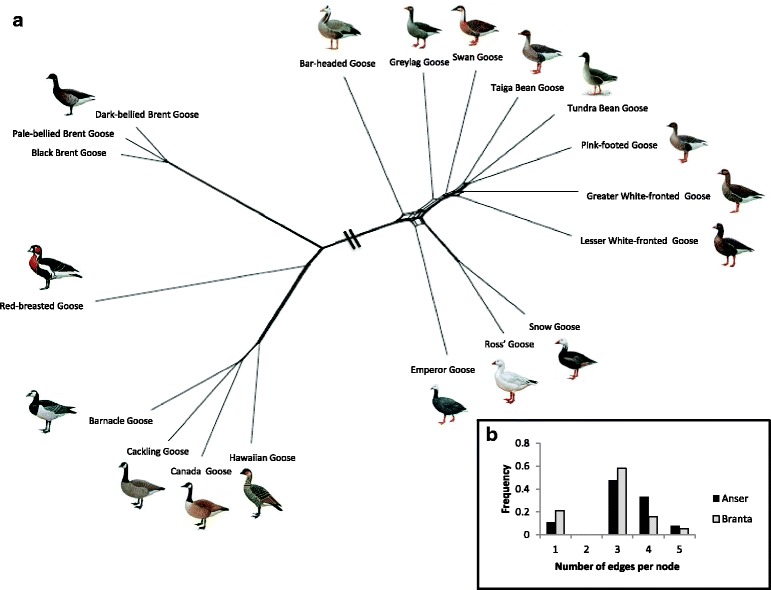
**a** Neighbour-joining Network of the True Geese using the ordinary least squares method (with default settings) in SplitsTree version 4.1.4.2 [15], based on genetic distances. **b** The comparison of degree distributions indicates that the *Anser-*network is more complex compared to the *Branta*-network as it contains relatively more nodes with four and five edges. Drawings used with permission of Handbook of Birds of the World [128]

The phylogenetic neighbour-joining network (Fig. 3) based on genetic distances uncovered two main clades that corresponded to the genera *Anser* and *Branta*. Within these clades, the relationships correspond to previous phylogenetic analyses [12]. The comparison of degree distributions revealed that the *Anser*-network was more complex compared to the *Branta-*network (Poisson regression, SD = 0.1908, *z-value* = −5.08, *p-value* < 0.001), because the *Anser*-network contains more nodes with four or five edges compared to the *Branta*-network. The complexity of the networks was consistent with the suggestion that the evolutionary history of the *Anser*-clade is more heavily influenced by rapid diversification and hybridization compared to the *Branta*-clade.

**Fig. 4:**
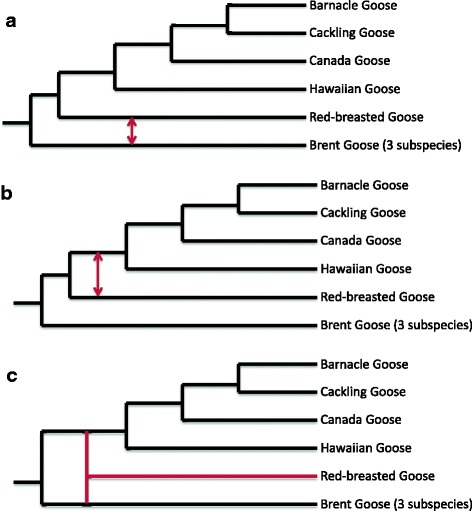
Hybridization networks for the genus *Branta* based on the Autumn algorithm [49] in Dendroscope version 3.4.4 [50]. Network **a** suggests hybridization between Red-breasted Goose and Brent Goose, network **b** between Red-breasted Goose and the ancestor of the White-cheeked Geese. Network **c** suggests a hybrid origin for the Red-breasted Goose

We combined 3558 gene trees into hybridization networks for both genera. These networks attempt to reconstruct a phylogenetic tree with the fewest amount of hybridization events [15, 48]. Hybridization network analyses of the genus *Anser* did not result in most likely scenarios, underlining the complexity of introgression and incomplete lineage sorting among *Anser* species. In the genus *Branta*, the hybridization network analyses recovered three (not mutually exclusive) scenarios, indicating hybridization events between the Red-breasted Goose and the ancestor of the White-cheeked Geese (i.e. Hawaiian Goose, Canada Goose, Cackling Goose and Barnacle Goose) and between Red-breasted Goose and Brent Goose (Fig. 4a-b). In addition, one hybridization network (Fig. 4c) suggested a hybrid origin for the Red-breasted Goose. The network suggesting a hybrid origin for this species should not be regarded as definitive proof for hybrid speciation, but rather as a possible scenario that can serve as a starting point for further research.

### Demographic analysis

**Fig. 5:**
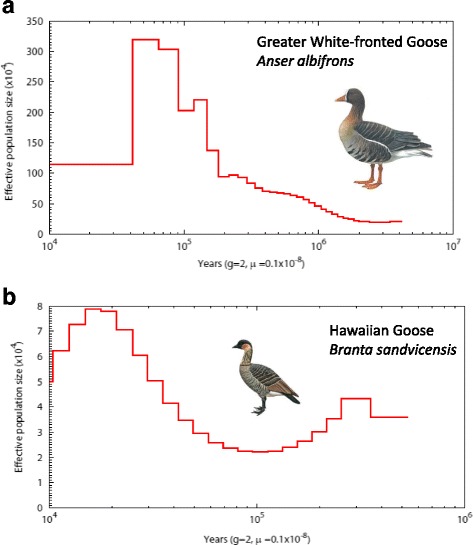
Examples of two general demographic patterns for the True Geese based on PSMC analyses. **a** Steady population increase followed by dramatic expansion which suggests population subdivision, as illustrated by Greater White-fronted Goose. **b** Population bottleneck after island colonization, as illustrated by Hawaiian Goose

We reconstructed historical effective populations sizes (N_e_) for all goose species using the pairwise sequentially Markovian coalescent (PSMC) approach over a range from 1 to 10 million years ago until about 10,000 years ago. Most *Anser* species (Greater White-fronted Goose, Lesser White-fronted Goose, Tundra Bean Goose, Taiga Bean Goose, Pink-footed Goose, Swan Goose, Greylag Goose, Bar-headed Goose, Snow Goose, and Ross’ Goose) and several *Branta* species (Canada Goose, Cackling Goose, Red-breasted Goose, Pale-bellied Brent Goose and Black Brent Goose) show a steady population increase followed by a dramatic expansion, which suggests population subdivision and occasional gene flow, leading to higher levels of heterozygosity and consequently higher estimates of N_e_ [51, 53]. Four species (Hawaiian Goose, Emperor Goose, Barnacle Goose and Dark-bellied Brent Goose) show clear signs of a bottleneck. Figure 5 shows these two patterns as illustrated by Greater White-fronted Goose and Hawaiian Goose (for other species, see Additional file 4: Figure S2).

## Discussion

### General patterns of introgression

Interspecific gene flow is an important aspect in avian speciation [54]. Based on hybridization networks and D-statistics, calculated from genome-wide data, we found indications for high levels of interspecific gene flow between several goose species. D-statistics allowed us to confidently discriminate between incomplete lineage sorting and interspecific gene flow. The significant D-statistics varied from 0.07 to 0.17, which is slightly higher compared to analyses on recent radiations, such as Darwin’s Finches (0.004–0.092; [55]) and butterflies of the genera *Heliconius* (0.04; [56]) and *Papilio* (0.04; [57]). These values do fall within the range of studies on other hybridizing species, such as pigs (0.11–0.23; [58]), bears (0.04–0.46; [59, 60]) and *Xiphophorus* fish (0.03–0.56; [61]).

A significant D-statistic does not necessarily indicate introgression between the species from which the genomes are being compared. There might have been gene flow with an extinct (not sampled) population or the signal might be a remnant from an older hybridization event [33, 62]. The latter possibility is probably the case for hybridization between Red-breasted Goose and three other species (Hawaiian Goose, Canada Goose and Cackling Goose). The hybridization network analysis supported the notion that significant D-statistics were caused by an ancient hybridization event between Red-breasted Goose and the ancestor of these three species. Many of the significant D-statistics in the *Anser*-clade can probably be explained in the same way, but the complexity of introgression patterns in this clade did not allow us to pinpoint putative hybridization events. In addition, the D-statistic only captures asymmetric gene flow [33]. Because we did not quantify symmetric gene flow, we are probably underestimating the amount of gene flow between the some goose species.

When did this gene flow occur? Further analyses, based on the divergence times of 3558 gene trees, indicated that this gene flow was largely due to ancient hybridization during the diversification of these species. Ancient gene flow has been reported for a variety of taxa [46, 63, 64], including several bird groups [55, 65–68]. For instance, Fuchs et al. [65] attributed a conflicting pattern between several loci to ancient hybridization between members of the woodpecker genus *Campephilus* and the melanerpine lineage (*Melanerpes* and *Sphyrapicus*). The increasing number of studies reporting ancient gene flow during species diversification [69] shows that the speciation process is often more complex than, for example, the classical allopatric speciation model [70, 71].

In the allopatric speciation model, populations become geographically isolated and diverge by genetic drift and/or differential selection pressures, resulting in intrinsic reproductive isolation due to the accumulation of Dobzhansky-Muller incompatibilities [72, 73]. This speciation model predicts that the distribution of interspecific divergence is largely determined by a single, shared species split [74]. But speciation is often more complex: in some cases, speciation may advance by divergent ecological or sexual selection in the face of ongoing gene flow [75], while, in other cases, allopatrically diverging populations may come into secondary contact and hybridize before reproductive isolation is complete [2]. These more complex speciation models predict that interspecific divergence varies considerably across the genome [6, 7], because some genomic regions reflect the initial species split time, whereas others indicate more recent genetic exchange [11, 13, 76].

With regard to the evolutionary history of geese, we found support for a complex speciation model with high levels of gene flow during species diversification. It is, however, not possible to determine whether this gene flow is the outcome of (repeated) secondary contact or divergence-with-gene-flow. ABC modelling based on multiple samples per species allows for the comparison of several scenarios that differ in the amount and timing of gene flow and can thus be used to confidently discriminate between divergence-with-gene-flow and secondary contact [77–79]. For example, Nadachowska-Brzyska et al. [80] compared 15 models (with different patterns and levels of gene flow) to assess the demographic history of Pied Flycatcher (*Ficedula hypoleuca*) and Collared Flycatcher (*Ficedula albicollis*). Whole genome re-sequencing data from 20 individuals supported a recent divergence with unidirectional gene flow from Pied Flycatcher into Collared Flycatcher after the Last Glacial Maximum, indicating that the hybrid zone between these species is a secondary contact zone. In this study, we were unable to perform an ABC modelling exercise because only one individual per species was sampled, while multiple samples per species are required.

Next to evidence for ancient gene flow, our results suggest low levels of recent gene flow, which can be explained in three ways. First, the D-statistic analysis may be unable to detect recent gene flow. Indeed, the D-statistic was developed to detect ancient gene flow and to estimate the extent of archaic ancestry in the genomes of extant populations [33]. The detection and quantification of recent gene flow warrants a population genomic approach whereby multiple individuals of one population are sequenced [3, 9, 81, 82]. Second, the relative rarity of goose hybrids diminishes the opportunity for backcrossing and introgression, leading to absence or low levels of recent gene flow [19]. Third, there may be little recent gene flow because of strong intrinsic and/or extrinsic selection against goose hybrids. Although most goose hybrids are viable and fertile [19], second generation hybrids or backcrosses may be impaired by genetic incompatibilities [83, 84], or hybrids might be ecologically maladapted (e.g., intermediate beak morphology) or unable to find a mate [73]. To answer these questions, field observations are needed, which is challenging given the relative rarity of hybrids [19] and the difficulty of identifying certain hybrids [85]. Strong selection against hybrids might also suggest that the diversification of the True Geese was partly driven by reinforcement [86].

### Demographic patterns

The reconstruction of historical effective populations sizes (N_e_) for all goose species using the pairwise sequentially Markovian coalescent (PSMC) approach indicated two main patterns. First, most species showed a steady increase during the Pliocene and Pleistocene followed by population subdivision (apparent as a dramatic increase in N_e_) during the Last Glacial Maximum (LGM, about 110,000 to 12,000 years ago). The increase in population size during the Pliocene and Pleistocene can be explained by a global cooling trend which resulted in the formation of a circumpolar tundra and the emergence of temperate grasslands [87–89]. The tundra habitat acted as breeding ground [90], whereas the grasslands served as wintering grounds where mate choice occurred [91], enabling goose populations to proliferate. In addition, the climatic fluctuations during the Pliocene and Pleistocene might have instigated range expansions and shifts. This combination of large N_e_ and occasional range shifts might have facilitated contact between the diverging goose species, resulting in the establishment of numerous hybrids zones and consequent gene flow [92, 93].

During the LGM, many plant and animal populations were subdivided into separate refugia by the ice sheets that expanded from the north [94, 95]. This population subdivision has been described for several goose species [96] and the genetic signature of this subdivision has been uncovered for certain species, such as Pink-footed Goose [97], Bean Goose [98], Greater White-fronted Goose [99, 100], Canada Goose [101], and Snow Goose [26, 102].

Four species show a decrease in Ne and a consequent genetic bottleneck in the PSMC analyses, which suggests island colonization. Indeed, these four goose species have colonized island habitats: the Hawaiian Goose reached the Hawaiian archipelago [103], the Emperor Goose settled on the Aleutian Islands [104], and the Barnacle Goose and the Dark-bellied Brent Goose established populations on arctic islands in the North Atlantic, such as Spitsbergen and Novaya Zemlya [105]. It is well-established that island colonization leads to a reduction in heterozygosity and N_e_ [106], and that island populations have lower levels of genetic variation compared to mainland species [107]. Genetic bottlenecks following island colonization have been documented for numerous other bird species (e.g., [108, 109]). However, further analyses are warranted to confirm these scenarios of island colonization. For instance, comparing the genetic diversity of these four goose species with closely related mainland populations.

### Comparing Anser and Branta

There is a striking contrast in the patterns of introgression between the two genera. As hypothesised, the general network analysis showed that the *Anser*-network is more complex than the *Branta*-network and D-statistics were slightly (although not significantly) higher in the *Anser*-clade. While high levels of gene flow hindered the precise reconstruction of hybridization events in the *Anser*-clade*,* it was possible to pinpoint several putative hybridization events within *Branta*-clade. The hybridization network analyses provided evidence for gene flow between the Red-breasted Goose and the ancestor of the White-cheeked Geese (i.e. Hawaiian Goose, Canada Goose, Cackling Goose and Barnacle Goose), between Red-breasted Goose and Brent Goose, and between Canada Goose and Cackling Goose. Past gene flow between the latter two species has been reported previously [23]. What factors can explain the differential introgression patterns between *Anser* and *Branta*? We will consider three possible factors: (1) macro-evolutionary dynamics, (2) morphological and behavioural differences, and (3) demographic dynamics.

First, these patterns of introgression were reconstructed by comparing the genomes of modern, extant species. The ancestors of these modern species may have interbred with unknown extinct species. It might thus be possible that the evolutionary history of the *Branta*-clade was as influenced by hybridization as much as the diversification of the *Anser*-clade, but that many *Branta*-species have become extinct. For example, the Hawaiian radiation of *Branta* geese consisted of at least three species, of which only the Hawaiian Goose remains today [103]. The different introgression patterns (as observed by comparing extant genomes) could then be attributed to differences in extinction rates between the genera. Unfortunately, the fossil record for geese is currently still too sparse to test this hypothesis [110, 111].

Second, differential introgression patterns may be explained by differences in behaviour [112, 113]. Although the behaviour of extant species does not necessarily correspond to the ancestral behaviour, we can speculate about possible differences between the genera. Pair formation, involving several pre-copulatory displays, and copulation vary little between the species and the genera [90, 114], which can explain the frequent occurrence of hybridization on the species level, but does not clarify the differences in introgression patterns between the genera. Are there differences in certain behaviours that lead to hybridization, such as interspecific nest parasitism or forced extra-pair copulations [115]? These behaviours have been observed in both genera, but the relative contribution of each behaviour to the occurrence of goose hybrids remains to be quantified [19].

Mate choice in waterfowl is largely determined by sexual imprinting [116]. *Anser* species are morphologically more similar compared to *Branta* species, which might increase the probability of heterospecific mate choice. Based on this reasoning, we expect more *Anser* hybrids compared to *Branta*. This expectation remains to be tested, but will be challenging because hybrids between morphologically similar species are difficult to identify [85] and many goose hybrids are probably of captive origin [19].

Third, differences in demographic dynamics, mediated by a particular biogeographical and climatic context, might determine the frequency of interspecific interactions, possibly leading to introgressive hybridization. The *Anser*-clade has a largely Eurasian distribution (with the exception of Snow Goose and Ross’ Goose). The open tundra landscape of Eurasia during the Pleistocene allowed for large effective population sizes and the climatic fluctuations during the Pliocene and Pleistocene might have instigated range expansions and shifts. In contrast to the *Anser*-clade, the *Branta* species are more widely distributed across the Northern Hemisphere: Canada Goose and Cackling Goose in North America, Hawaiian Goose on the Hawaiian islands, Barnacle Goose and Red-breasted Goose in Eurasia, and the circumpolar Brent Goose. This distribution limits the frequency of interspecific contact, although several species could achieve large effective population sizes.

The demographic differences between the genera might also lead to other speciation histories. The diversification of the *Branta*-clade was more gradual compared to the *Anser*-clade, which can be considered an adaptive radiation [12]. During an adaptive radiation the frequency of interspecific interactions increases, enhancing the probability of introgressive hybridization [117]. Moreover, as the radiation progresses, occasional hybridization could facilitate further ecological diversification [118]. Possibly, the diversification in beak morphology among *Anser* species was driven by hybridization, comparable to the radiation of Darwin’s Finches on the Galapagos Islands [55, 119].

### A hybrid origin for the Red-breasted Goose?

The hybridization network analysis also suggested a possible alternative scenario in which the Red-breasted Goose is a hybrid species between the ancestors of the White-cheeked Geese and the Brent Goose. If so, the distinct morphology of this species, which is not intermediate between its putative parents, might be the outcome of transgressive segregation [120]. But indisputably demonstrating hybrid speciation is challenging and often the most likely scenario for the observed genomic pattern is introgressive hybridization [121]. To our knowledge, five bird species have been proposed to have hybrid origins: the Italian Sparrow (*Passer italiae*, [122]), the Audubon’s Warbler (*Setophaga auduboni*, [123]), the Genovesa Mockingbird (*Mimus parvulus bauri*, [124]), the Hawaiian Duck (*Anas wylvilliana*, [125]) and a recent lineage of Darwin’s finches on Daphne Major (referred to as ‘Big Bird’, [126]). However, the hybrid origin of these putative cases has not been unequivocally established [121]. Also, in the case of the Red-breasted Goose, the most parsimonious explanation seems to involve separate hybridization events between the Red-breasted Goose and the ancestor of the White-cheeked Geese and between Red-breasted Goose and Brent Goose. If the Red-breasted Goose is a hybrid species, one would expect significantly higher values for D-statistics. For example, a recent genomic study of the Italian Sparrow, a hybrid species between House Sparrow (*Passer domesticus*) and Spanish Sparrow (*Passer hispaniolensis*), uncovered D-statistics over 50% [127]. The highest value for Red-breasted Goose in our analysis was about 15% (even some *Anser* species displayed higher D-statistics). Hence, a hybrid origin for the Red-breasted Goose seems unlikely.

## Conclusions

Using genomic datasets and modern analysis tools, such as the D-statistic and PSMC analysis, in combination with network analyses based on gene tree discordance, we were able to determine patterns of introgressive hybridization in the True Geese. High levels of ancient gene flow suggest a scenario of divergence-with-gene-flow. We found indications for low levels of recent gene flow, but the quantification of this recent gene flow warrants a population genomic approach whereby multiple individuals of one population are sequenced. The reconstruction of historical effective population sizes indicates that most species showed a steady increase during the Pliocene and Pleistocene followed by population subdivision during the Last Glacial Maximum about 110,000 to 12,000 years ago. The combination of large effective population sizes and occasional range shifts might have facilitated contact between diverging goose species, resulting in the establishment of numerous hybrid zones and consequent gene flow. Our approach, based on genome-wide phylogenetic incongruence and network analyses, will be a useful procedure to reconstruct the complex evolutionary histories of many naturally hybridizing species groups.

## Additional files

Additional file 1: Table S1. Sampled goose species and sampling location. (DOCX 15 kb)

Additional file 2: Table S2. Mapping results of all goose samples to Mallard genome (version 73) using SMALT. (DOCX 20 kb)

Additional file 3: Table S3. D-statistics for all combinations of three species per genus. Significant D-statistics (Z-score > 4), suggesting gene flow between P2 and P3, are indicated in bold and colored in yellow. The outgroup for *Branta* was a consensus sequence based on all *Anser* species. Similarly, the outgroup for *Anser* was a consensus sequence based on all *Branta* species. (DOCX 19 kb)

Additional file 4: Figure S1. Distribution of gene tree divergence times for all goose species. All distributions show a single peak, indicating gene flow during divergence. The divergence time of several gene trees was close to zero, suggesting low levels of recent gene flow between certain species. Final three figures represent the three subspecies of Brent Goose, which is depicted in the lower right panel. (ZIP 2715 kb)

Additional file 5: Figure S2. Estimates of historical effective population sizes for all goose species, based on a PSMC analysis. Final three figures represent the three subspecies of Brent Goose, which is depicted in the lower right panel. (ZIP 766 kb)

## Abbreviations

ABC Modelling: Approximate Bayesian Computation Modelling
Bp: Base pairs
GATK: Genome Analysis Toolkit
GTR: General Time-reversible
IUPAC: International Union of Pure and Applied Chemistry
LGM: Last Glacial Maximum
N_e_: Effective Population Size
PSMC: Pairwise Sequentially Markovian Coalescent
SD: Standard Deviation
