# Supplementary material for: A history of hybrids? Genomic patterns of introgression in the True Geese
Source: BMC Evol Biol. 2017 Aug 22;17:201. doi: 10.1186/s12862-017-1048-2 (PMC5568201; doi:10.1186/s12862-017-1048-2)
Supplement: Supplementary file 1 — Sampled goose species and sampling location. (DOCX 15 kb) [file 12862_2017_1048_MOESM1_ESM.docx]

**Table S1: Sampled goose species and sampling location**

| **Species** | | **Sampling Location** |
| --- | --- | --- |
| **Common Name** | **Scientific Name** |  |
| **GENUS ANSER** |  |  |
| Greater White-fronted Goose | *A. albifrons* | Collection Müskens |
| Lesser White-fronted Goose | *A. erythropus* | Collection Müskens |
| Greylag Goose | *A. anser* | Collection Brenders |
| Swan Goose | *A. cygnoides* | Avifauna (Alphen aan de Rijn, NL) |
| Pink-footed Goose | *A. brachyrhynchus* | NIOO |
| Taiga Bean Goose | *A. fabalis* | Collection Müskens |
| Tundra Bean Goose | *A. serrirostris* | Collection Müskens |
| Bar-headed Goose | *A. indicus* | Ouwehands Zoo (Rhenen, NL) |
| Emperor Goose | *A. canagica* | Collection Meinen |
| Ross’ Goose | *A. rossii* | Avifauna |
| Snow Goose | *A. caerulescens* | Collection Meinen |
|  |  |  |
| **GENUS BRANTA** |  |  |
| Dark-bellied Brent Goose | *B. bernicla bernicla* | Collection Meinen |
| Pale-bellied Brent Goose | *B. b. hrota* | Collection Brenders |
| Black Brent Goose | *B. b. nigricans* | Collection Meinen |
| Canada Goose | *B. canadensis* | NIOO |
| Cackling Goose | *B. hutchinsii* | Avifauna |
| Barnacle Goose | *B. leucopsis* | Collection Müskens |
| Red-breasted Goose | *B. ruficollis* | Avifauna |
| Hawaii Goose | *B. sandvicensis* | Collection Meinen |
