# Supplementary material for: A history of hybrids? Genomic patterns of introgression in the True Geese
Source: BMC Evol Biol. 2017 Aug 22;17:201. doi: 10.1186/s12862-017-1048-2 (PMC5568201; doi:10.1186/s12862-017-1048-2)
Supplement: Supplementary file 2 — Mapping results of all goose samples to Mallard genome (version 73) using SMALT. (DOCX 20 kb) [file 12862_2017_1048_MOESM2_ESM.docx]

| **Species** | **N Reads** | **Reads Mapped** | **Reads Properly Paired** | **N reads**  **(after processing*)** | **Average Depth**  **of Coverage** |
| --- | --- | --- | --- | --- | --- |
| Swan Goose (*Anser cygnoides*) | 248,073,165 | 246,361,460 (99.31%) | 171,741,052 (69.23%) | 167,787,462 | 15.5777 |
| Lesser White-fronted Goose (*Anser erythropus*) | 239,825,881 | 238,147,100 (99.30%) | 151,282,166 (63.08%) | 147,604,596 | 13.6891 |
| Greater White-fronted Goose (*Anser albifrons*) | 209,540,538 | 208,157,570 (99.34%) | 165,243,668 (78.86)% | 168,279,164 | 15.5885 |
| Pink-footed Goose (*Anser brachyrhynchus*) | 243,037,139 | 240,339,427 (98.89%) | 180,819,631 (74.40%) | 185,068,118 | 17.138 |
| Greylag Goose (*Anser anser*) | 180,263,790 | 178,857,732 (99.22%) | 134,044,154 (74.36%) | 137,523,408 | 12.8095 |
| Taiga Bean Goose (*Anser fabalis*) | 234,490,156 | 231,934,213 (98.91%) | 175,609,678 (74.89%) | 182,498,873 | 16.9499 |
| Bar-headed Goose (*Anser indicus*) | 178,281,121 | 176,034,779 (98.74%) | 132,017,170 (74.05%) | 134,342,616 | 12.5142 |
| Tundra Bean Goose (*Anser serrirostris*) | 237,129,371 | 234,141,541 (98.74%) | 177,040,788 (74.66%) | 181,589,528 | 16.8361 |
| Snow Goose (*Anser caerulescens*) | 151,597,232 | 150,642,169 (99.37%) | 111,151,091 (73.32%) | 113,220,052 | 10.5805 |
| Ross’ Goose (*Anser rossii*) | 146,841,711 | 145,681,661 (99.21%) | 110,982,965 (75.58%) | 115,445,482 | 10.7958 |
| Emperor Goose (*Anser canagicus*) | 152,586,156 | 151,579,087 (99.34%) | 115,370,393 (75.61%) | 114,487,545 | 10.6968 |
| Hawaii Goose (*Branta sandvicensis*) | 149,320,696 | 148,230,655 (99.27%) | 113,334,408 (75.90%) | 116,924,557 | 10.9345 |
| Canada Goose (*Branta canadensis*) | 149,260,299 | 148,051,291 (99.19%) | 106,661,410 (71.46%) | 106,452,024 | 9.93374 |
| Red-breasted Goose (*Branta ruficollis*) | 145,708,845 | 144,324,611 (99.05%) | 99,999,980 (68.63%) | 105,189,385 | 9.84807 |
| Cackling Goose (*Branta hutchinsii*) | 146,420,070 | 145,424,414 (99.32%) | 110,883,919 (75.73%) | 116,028,146 | 10.8286 |
| Dark-bellied Brent Goose (*Branta bernicla bernicla*) | 149,595,960 | 148,264,556 (99.11%) | 106,437,526 (71.15%) | 108,789,885 | 10.1684 |
| Pale-bellied Brent Goose (*Branta bernicla hrota*) | 149,880,011 | 148,725,935 (99.23%) | 107,643,824 (71.82%) | 110,483,376 | 10.3212 |
| Black Brent Goose (*Branta bernicla nigricans*) | 149,004,169 | 147,782,335 (99.18%) | 105,867,462 (71.05%) | 108,191,843 | 10.0909 |
| Barnacle Goose (*Branta leucopsis*) | 151,975,535 | 150,942,101 (99.32%) | 117,781,040 (77.50%) | 118,981,896 | 11.0964 |

* Processing includes removal of duplicate sequences (Samtools) and realignment (GATK)
