# Supplementary material for: A history of hybrids? Genomic patterns of introgression in the True Geese
Source: BMC Evol Biol. 2017 Aug 22;17:201. doi: 10.1186/s12862-017-1048-2 (PMC5568201; doi:10.1186/s12862-017-1048-2)
Supplement: Supplementary file 3 — D-statistics for all combinations of three species per genus. Significant D-statistics (Z-score > 4), suggesting gene flow between P2 and P3, are indicated in bold and colored in yellow. The outgroup for Branta was a consensus sequence based on all Anser species. Similarly, the outgroup for Anser was a consensus sequence based on all Branta species. (DOCX 19 kb) [file 12862_2017_1048_MOESM3_ESM.docx]

**Table S3. D-statistics for all combinations of three species per genus. Significant D-statistics (Z-score > 4), suggesting gene flow between P2 and P3, are indicated in bold and colored in yellow. Outgroup for *Branta* was a consensus sequence based on all *Anser* species. Similarly, the outgroup for *Anser* was a consensus sequence based on all *Branta* species.**

| **Species 1** | **Species 2** | **Species 3** | **D-statistic** | **Z-score** |
| --- | --- | --- | --- | --- |
| Barnacle Goose | Cackling Goose | Canada Goose | **0.08728** | **4.32154** |
| Barnacle Goose | Cackling Goose | Red-breasted Goose | **0.14635** | **6.02174** |
| Canada Goose |  |  | 0.04229 | 2.02795 |
| Hawaii Goose |  |  | 0.05084 | 2.21083 |
| Barnacle Goose | Cackling Goose | Black Brent | **0.09358** | **3.5156** |
| Canada Goose |  |  | 0.02062 | 0.84617 |
| Hawaii Goose |  |  | 0.04735 | 1.99313 |
| Red-breasted Goose |  |  | NA | NA |
| Barnacle Goose | Canada Goose | Red-breasted Goose | **0.10018** | **4.03718** |
| Cackling Goose |  |  | NA | NA |
| Hawaii Goose |  |  | 0.01082 | 0.4553 |
| Barnacle Goose | Hawaii Goose | Red-breasted Goose | **0.0937** | **4.19242** |
| Cackling Goose |  |  | NA | NA |
| Canada Goose |  |  | NA | NA |
| Barnacle Goose | Black Brent | Red-breasted Goose | **0.07249** | **3.02167** |
| Cackling Goose |  |  | 0.00681 | 0.29991 |
| Canada Goose |  |  | 0.02213 | 0.99801 |
| Hawaii Goose |  |  | 0.04413 | 1.91339 |

| **Species 1** | **Species 2** | **Species 3** | **D-statistic** | **Z-score** |
| --- | --- | --- | --- | --- |
| Lesser WF | Greater WF | Pink-footed Goose | **0.12618** | **6.11657** |
| Tundra Bean Goose |  |  | 0.02723 | 1.33776 |
| Taiga Bean Goose |  |  | **0.08962** | **4.41287** |
| Lesser WF | Greater WF | Emperor Goose | 0.00458 | 0.19114 |
| Pink-footed Goose |  |  | **0.08824** | **3.70132** |
| Tundra Bean Goose |  |  | **0.07891** | **3.38305** |
| Taiga Bean Goose |  |  | **0.12782** | **5.45737** |
| Swan Goose |  |  | 0.06185 | 2.6321 |
| Greylag Goose |  |  | **0.07889** | **3.53742** |
| Snow Goose |  |  | 0.00213 | 0.0979 |
| Ross’ Goose |  |  | NA | NA |
| Lesser WF | Greater WF | Bar-headed Goose | 0.02266 | 0.91438 |
| Pink-footed Goose |  |  | 0.00384 | 0.17217 |
| Tundra Bean Goose |  |  | 0.04281 | 1.68711 |
| Taiga Bean Goose |  |  | **0.08341** | **3.47019** |
| Swan Goose |  |  | 0.02871 | 1.1552 |
| Greylag Goose |  |  | NA | NA |
| Greater WF | Lesser WF | Swan Goose | **0.11449** | **5.27904** |
| Pink-footed Goose |  |  | **0.1498** | **6.82075** |
| Tundra Bean Goose |  |  | **0.16054** | **8.14973** |
| Taiga Bean Goose |  |  | **0.17324** | **8.38472** |
| Greater WF | Lesser WF | Greylag Goose | **0.10818** | **5.26884** |
| Pink-footed Goose |  |  | **0.08162** | **3.5794** |
| Tundra Bean Goose |  |  | **0.0973** | **4.64227** |
| Taiga Bean Goose |  |  | **0.08177** | **3.70011** |
| Swan Goose |  |  | **0.09312** | **4.06701** |
| Greater WF | Lesser WF | Ross’ Goose | **0.10396** | **5.32508** |
| Pink-footed Goose |  |  | **0.11974** | **5.42133** |
| Tundra Bean Goose |  |  | **0.10336** | **4.68194** |
| Taiga Bean Goose |  |  | **0.08314** | **3.93028** |
| Swan Goose |  |  | **0.1038** | **3.98444** |
| Greylag Goose |  |  | 0.01148 | 0.53256 |
| Snow Goose |  |  | NA | NA |
| Emperor Goose |  |  | **0.10199** | **4.5106** |
| Greater WF | Lesser WF | Snow Goose | **0.16526** | **7.33651** |
| Pink-footed Goose |  |  | **0.17361** | **7.03194** |
| Tundra Bean Goose |  |  | **0.15702** | **7.05339** |
| Taiga Bean Goose |  |  | **0.13653** | **6.22385** |
| Swan Goose |  |  | **0.15579** | **6.52462** |
| Greylag Goose |  |  | **0.08565** | **4.05429** |
| Ross’ Goose |  |  | 0.05399 | 2.29222 |
| Emperor Goose |  |  | **0.14437** | **6.00712** |
| Greater WF | Lesser WF | Emperor Goose | NA | NA |
| Pink-footed Goose |  |  | **0.07564** | **3.16364** |
| Tundra Bean Goose |  |  | **0.0703** | **3.00986** |
| Taiga Bean Goose |  |  | **0.11995** | **5.41878** |
| Swan Goose |  |  | 0.05741 | 2.35746 |
| Greylag Goose |  |  | **0.07769** | **3.47207** |
| Ross’ Goose |  |  | NA | NA |
| Snow Goose |  |  | NA | NA |
| Greater WF | Pink-footed Goose | Bar-headed Goose | NA | NA |
| Lesser WF |  |  | 0.01861 | 0.74921 |
| Tundra Bean Goose |  |  | 0.04048 | 1.56798 |
| Taiga Bean Goose |  |  | **0.08173** | **3.43567** |
| Swan Goose |  |  | 0.02505 | 0.9412 |
| Greylag Goose |  |  | NA | NA |
| Greater WF | Greylag Goose | Ross’ Goose | **0.0867** | **3.71949** |
| Lesser WF |  |  | NA | NA |
| Pink-footed Goose |  |  | **0.10741** | **4.49622** |
| Tundra Bean Goose |  |  | **0.08642** | **3.4077** |
| Taiga Bean Goose |  |  | 0.06832 | 2.94125 |
| Swan Goose |  |  | **0.08417** | **3.04915** |
| Snow Goose |  |  | 0.03108 | 1.22365 |
| Emperor Goose |  |  | **0.16325** | **6.66969** |
| Greater WF | Greylag Goose | Snow Goose | **0.07162** | **3.05535** |
| Lesser WF |  |  | NA | NA |
| Pink-footed Goose |  |  | **0.09092** | **3.41593** |
| Tundra Bean Goose |  |  | 0.06595 | 2.68222 |
| Taiga Bean Goose |  |  | 0.04418 | 1.83175 |
| Swan Goose |  |  | 0.0588 | 2.22562 |
| Ross’ Goose |  |  | NA | NA |
| Emperor Goose |  |  | **0.13882** | **5.59049** |
| Greater WF | Tundra Bean Goose | Ross’ Goose | 0.00305 | 0.12683 |
| Lesser WF |  |  | NA | NA |
| Pink-footed Goose |  |  | 0.02867 | 1.23776 |
| Taiga Bean Goose |  |  | NA | NA |
| Swan Goose |  |  | 0.00034 | 0.01257 |
| Snow Goose |  |  | 0.00673 | 0.27436 |
| Emperor Goose |  |  | **0.07827** | **3.69219** |
| Greater WF | Tundra Bean Goose | Snow Goose | 0.00949 | 0.38375 |
| Lesser WF |  |  | NA | NA |
| Pink-footed Goose |  |  | 0.03252 | 1.24293 |
| Taiga Bean Goose |  |  | NA | NA |
| Swan Goose |  |  | 0.00617 | 0.24225 |
| Ross’ Goose |  |  | NA | NA |
| Emperor Goose |  |  | **0.07382** | **3.31701** |
| Greater WF | Taiga Bean Goose | Ross’ Goose | 0.02316 | 1.0319 |
| Lesser WF |  |  | NA | NA |
| Pink-footed Goose |  |  | 0.04881 | 2.25631 |
| Tundra Bean Goose |  |  | 0.02269 | 0.97237 |
| Swan Goose |  |  | 0.0202 | 0.77424 |
| Greylag Goose |  |  | NA | NA |
| Snow Goose |  |  | 0.00234 | 0.10261 |
| Emperor Goose |  |  | **0.13905** | **6.34584** |
| Greater WF | Taiga Bean Goose | Snow Goose | 0.03412 | 1.45166 |
| Lesser WF |  |  | NA | NA |
| Pink-footed Goose |  |  | 0.05813 | 2.39468 |
| Tundra Bean Goose |  |  | 0.02728 | 1.16662 |
| Swan Goose |  |  | 0.01747 | 0.77938 |
| Greylag Goose |  |  | NA | NA |
| Ross’ Goose |  |  | NA | NA |
| Emperor Goose |  |  | **0.13976** | **6.65546** |
| Greater WF | Greylag Goose | Bar-headed Goose | 0.01386 | 0.56111 |
| Lesser WF |  |  | 0.03647 | 1.45986 |
| Pink-footed Goose |  |  | 0.01757 | 0.73695 |
| Tundra Bean Goose |  |  | 0.05311 | 2.04765 |
| Taiga Bean Goose |  |  | **0.09** | **4.05902** |
| Swan Goose |  |  | 0.04084 | 1.55534 |
