# Supplementary figures and images for: A history of hybrids? Genomic patterns of introgression in the True Geese
Source: BMC Evol Biol. 2017 Aug 22;17:201. doi: 10.1186/s12862-017-1048-2 (PMC5568201; doi:10.1186/s12862-017-1048-2)

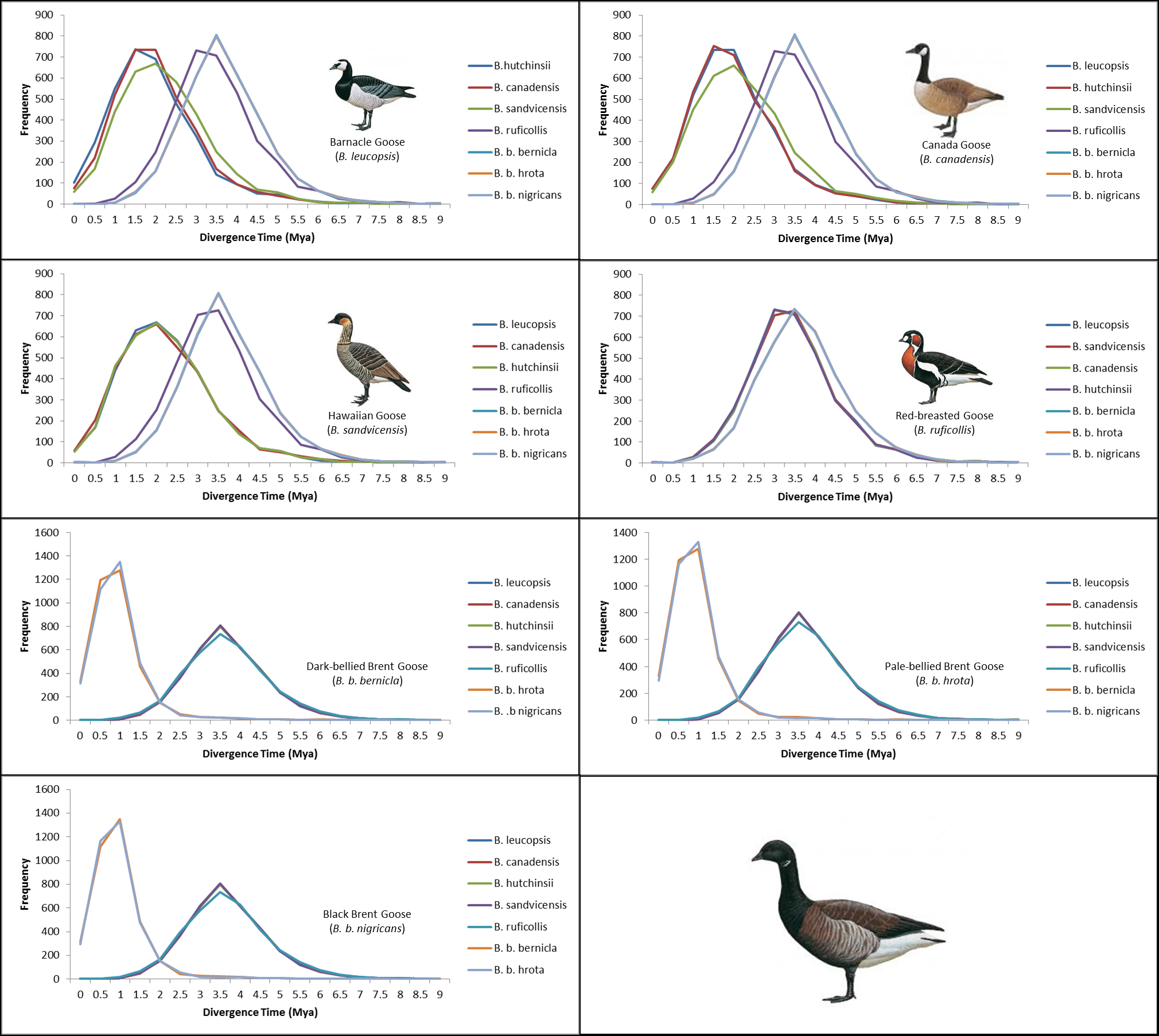

Supplement: Supplementary file 4 — Distribution of gene tree divergence times for all goose species. All distributions show a single peak, indicating gene flow during divergence. The divergence time of several gene trees was close to zero, suggesting low levels of recent gene flow between certain species. Final three figures represent the three subspecies of Brent Goose, which is depicted in the lower right panel. (ZIP 2715 kb) [file 12862_2017_1048_MOESM4_ESM.zip › S1 Divergence Times - Part 2.tif]

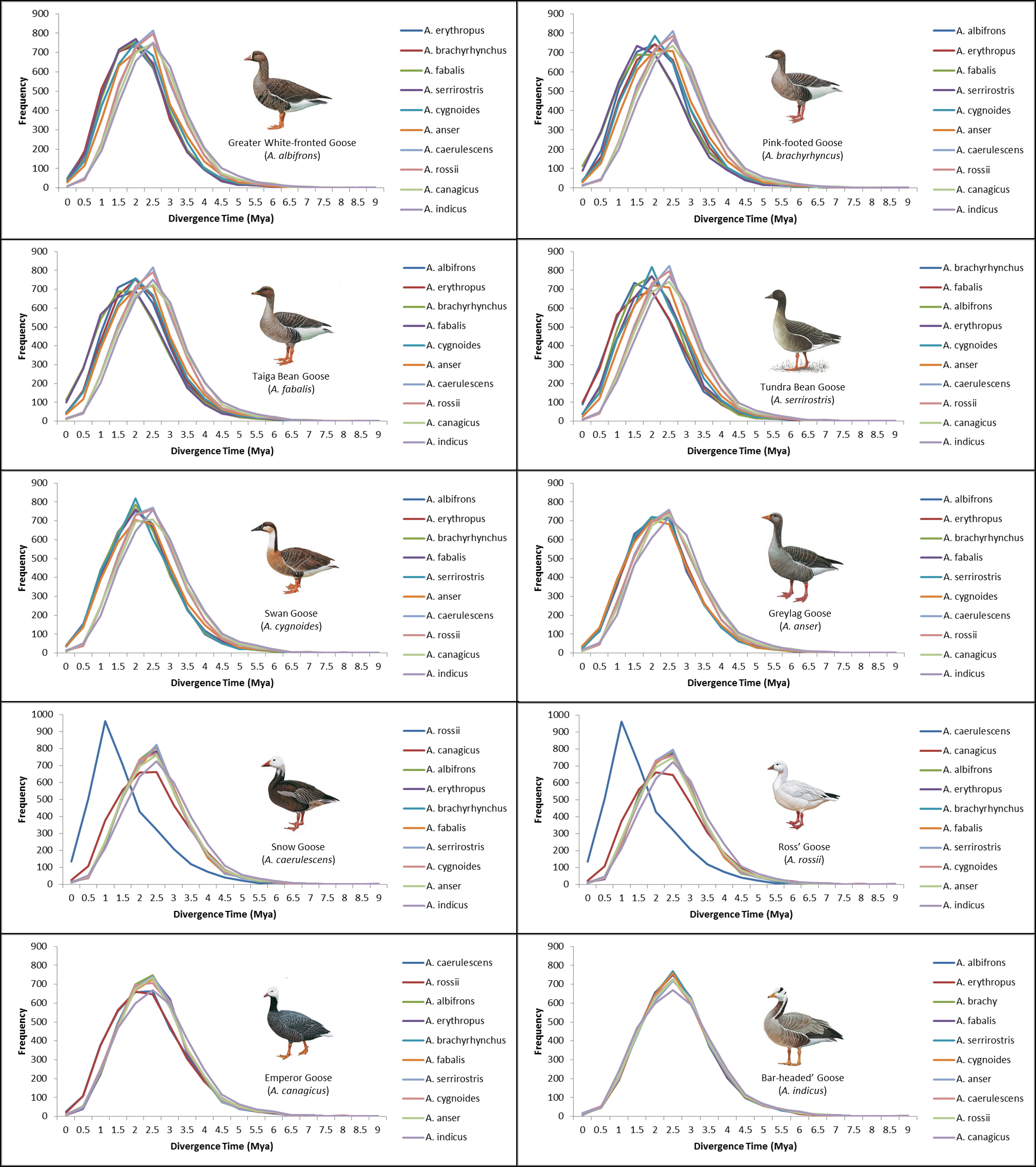

Supplement: Supplementary file 4 — Distribution of gene tree divergence times for all goose species. All distributions show a single peak, indicating gene flow during divergence. The divergence time of several gene trees was close to zero, suggesting low levels of recent gene flow between certain species. Final three figures represent the three subspecies of Brent Goose, which is depicted in the lower right panel. (ZIP 2715 kb) [file 12862_2017_1048_MOESM4_ESM.zip › S1 Divergence Times - Part 1.tif]

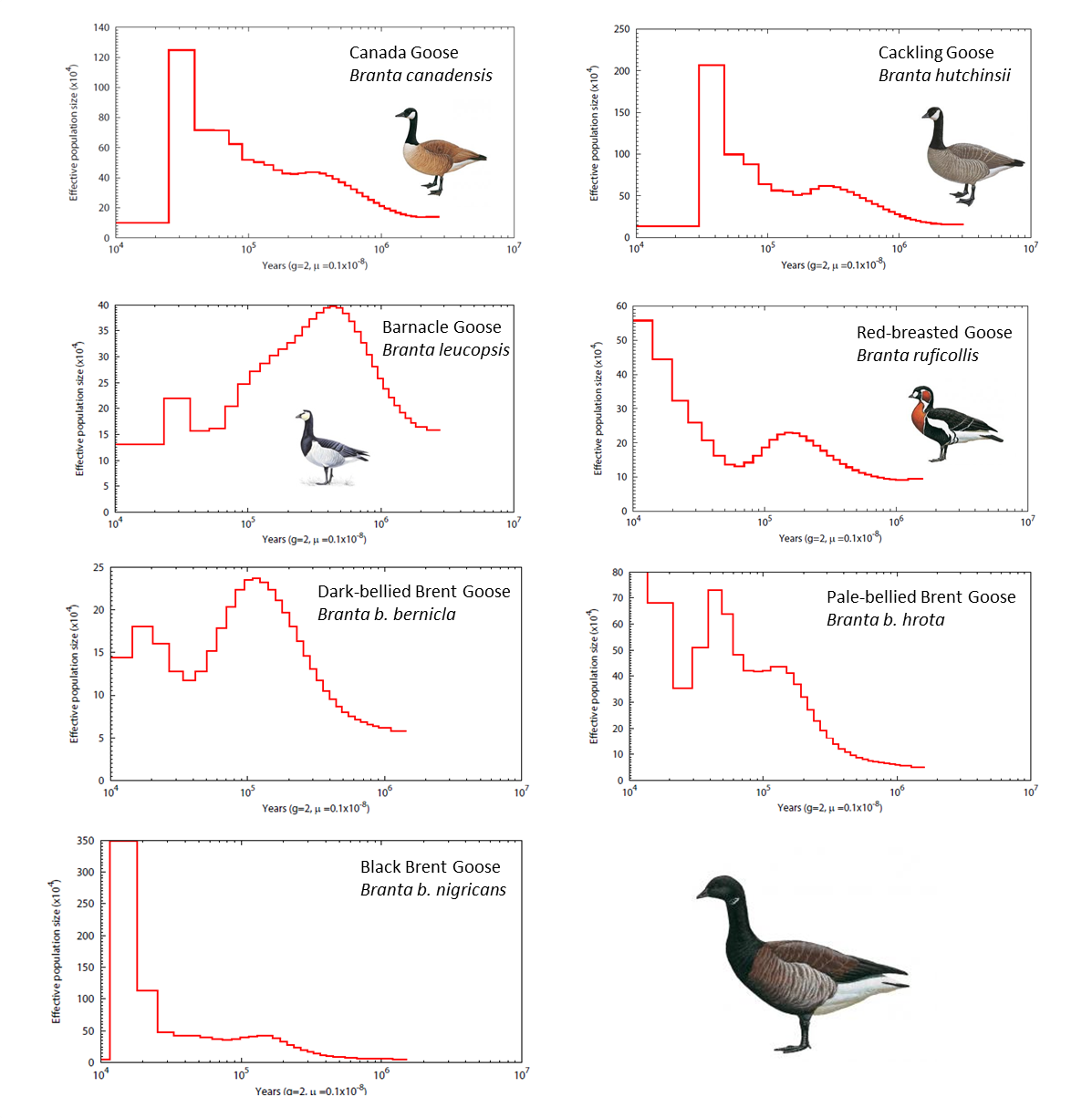

Supplement: Supplementary file 5 — Estimates of historical effective population sizes for all goose species, based on a PSMC analysis. Final three figures represent the three subspecies of Brent Goose, which is depicted in the lower right panel. (ZIP 766 kb) [file 12862_2017_1048_MOESM5_ESM.zip › S2 Demography - Part 2.tif]

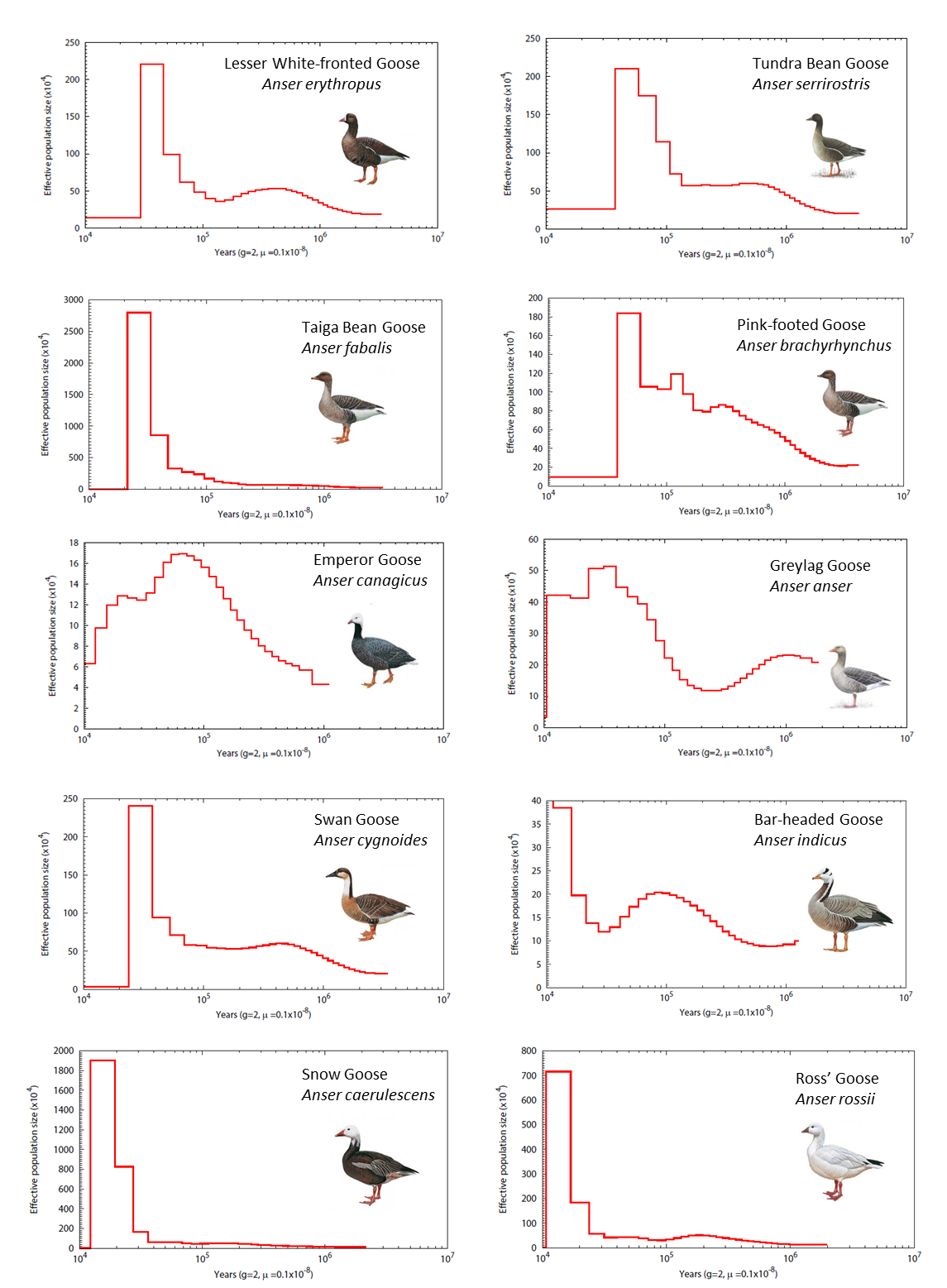

Supplement: Supplementary file 5 — Estimates of historical effective population sizes for all goose species, based on a PSMC analysis. Final three figures represent the three subspecies of Brent Goose, which is depicted in the lower right panel. (ZIP 766 kb) [file 12862_2017_1048_MOESM5_ESM.zip › S2 Demography - Part 1.tif]
